# LC–MS metabolomics and lipidomics in cerebrospinal fluid from viral and bacterial CNS infections: a review

**DOI:** 10.3389/fneur.2024.1403312

**Published:** 2024-08-05

**Authors:** Ontefetse Neo Plaatjie, A. Marceline Tutu van Furth, Martijn van der Kuip, Shayne Mason

**Affiliations:** ^1^Department of Biochemistry, Human Metabolomics, Faculty of Natural and Agricultural Sciences, North-West University, Potchefstroom, South Africa; ^2^Department of Pediatric Infectious Diseases and Immunology, Pediatric Infectious Diseases and Immunology, Amsterdam University Medical Center, Emma Children’s Hospital, Amsterdam, Netherlands

**Keywords:** lipidomics, metabolomics, meningitis, encephalitis, liquid chromatography mass spectrometer, neuroinflammation, cerebrospinal fluid, central nervous system

## Abstract

There is compelling evidence that a dysregulated immune inflammatory response in neuroinfectious diseases results in modifications in metabolic processes and altered metabolites, directly or indirectly influencing lipid metabolism within the central nervous system (CNS). The challenges in differential diagnosis and the provision of effective treatment in many neuroinfectious diseases are, in part, due to limited understanding of the pathophysiology underlying the disease. Although there are numerous metabolomics studies, there remains a deficit in neurolipidomics research to provide a comprehensive understanding of the connection between altered metabolites and changes in lipid metabolism. The brain is an inherently high-lipid organ; hence, understanding neurolipidomics is the key to future breakthroughs. This review aims to provide an integrative summary of altered cerebrospinal fluid (CSF) metabolites associated with neurolipid metabolism in bacterial and viral CNS infections, with a particular focus on studies that used liquid chromatography-mass spectrometry (LC–MS). Lipid components (phospholipids) and metabolites (carnitine and tryptophan) appear to be the most significant indicators in both bacterial and viral infections. On the basis of our analysis of the literature, we recommend employing neurolipidomics in conjunction with existing neurometabolomics data as a prospective method to enhance our understanding of the cross link between dysregulated metabolites and lipid metabolism in neuroinfectious diseases.

## Graphical Abstract

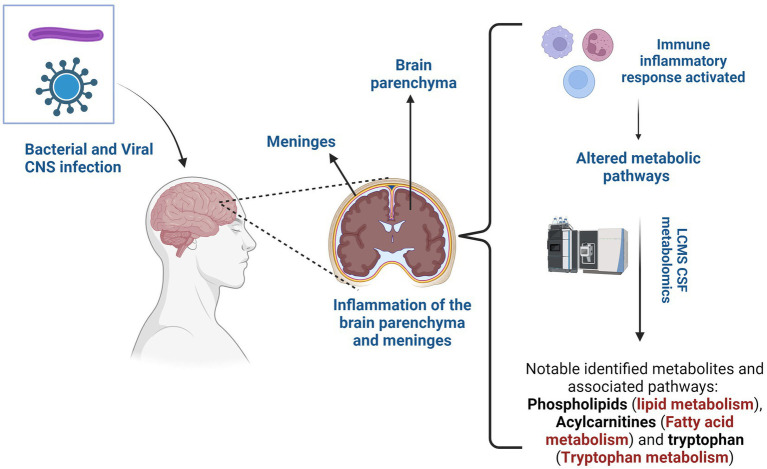

## 1 Introduction

Central nervous system (CNS) infections pose a significant public health challenge around the world due to their high morbidity and mortality (1). Without timely treatment, these infections can become life-threatening, particularly among children and immunocompromised adults, underscoring the urgency for prompt management. Bacteria, viruses, fungi, or parasites are capable of breaching the blood–brain barrier (BBB), a specialized protective structure designed to protect the CNS from microbial invasion, or can access the CNS through transneuronal routes that bypass the BBB (2). Viruses predominantly induce encephalitis, an inflammation of the brain parenchyma, while bacteria typically lead to meningitis – inflammation of the membranes surrounding the brain and spinal cord (3). Fungal and parasitic infections are relatively rare compared to viral and bacterial diseases and occur more frequently in immunocompromised individuals (4, 5).

Bacterial meningitis occurs with incidence rates ranging from approximately 0.9 per 100,000 individuals annually in high-income countries to 80 per 100,000 individuals annually in low-income countries (6). A wide range of bacteria can cause bacterial meningitis, and the causative bacteria vary depending on age (7). Similarly, viral CNS infection represents a significant burden on human health worldwide, with an annual incidence rate ranging from 0.26 to 17 cases per 100,000 individuals, often influenced by factors such as age distribution and vaccination status of the population (8). Even though both these conditions involve the inflammation of the CNS, they have distinct clinical presentations. Classic CSF indicators of bacterial meningitis include elevated white blood cells (WBC), primarily neutrophils, elevated protein levels and decreased glucose levels, as defined by Marais et al. (9). Viral meningitis presents with a mild to moderate increase in WBC, primarily lymphocytes, an elevated protein level and a normal glucose level within the CSF (10).

In both conditions, clinical presentations (fever, headache, and altered mental status) may not be adequate for a definitive diagnosis. Additional signs may include focal neurological signs and seizures (3). Neck pain or stiffness is a hallmark sign of meningitis that can occasionally be observed in encephalitis. Neurological dysfunction (cognitive function, behavior, consciousness or awareness) is a key clinical characteristic that prompts a further determination of encephalitis. Currently, there is no single test sufficient for an adequate diagnosis of meningitis or encephalitis; instead, a combination of multiple diagnostic tests is required for an accurate diagnosis. Although significant progress has been made in improving CNS infections diagnostics, the quest for rapid, accurate, and precise tests persists. Exploring novel biomarkers is emerging as imperative, which presents a promising avenue to not only aid in early diagnosis, but also provide clinicians with insight into disease progression and treatment strategies.

‘Omics’ approaches have transformed our understanding of disease processes, offering substantial opportunities to identify diagnostic biomarkers and facilitate early detection in neuroinfectious diseases (11). Neurometabolomics, an approach focused on comprehensive analysis of small metabolites, has emerged as a crucial tool to elucidate the altered metabolic pathways involved in CNS diseases. The CNS relies on tightly regulated energy metabolism to maintain its high metabolic demands (12). Understanding altered metabolic pathways in cerebrospinal fluid (CSF) could aid in a better understanding on the molecular mechanisms and diagnosis of CNS diseases and thus open avenues for targeted therapeutic interventions (13).

Neurolipidomics profiling is a powerful tool that allows for a comprehensive analysis of lipids and their associated pathways in the CNS. The field of neurolipidomics has received limited attention due to the historical absence of sufficiently sensitive analytical platforms and the complexity of the CNS lipidome, which makes lipidomics challenging (14, 15). However, due to recent advances in technology, the field of neurolipidomics is experiencing significant growth. For example, the study by Al-Mekhlafi et al. (16) identified elevated CSF phosphatidylcholines (PCs), a type of lipid, as a sensitive potential biomarker for bacterial meningitis with high precision in differentiating bacterial infections from viral infections in the CNS. The study also reported that while CSF lactate was found to be the highest biomarker with overall precision, the PC biomarkers offered high sensitivity and negative predictive value, important for minimizing false negative test results in cases of bacterial meningitis. These findings underscore the importance of CSF lipid profiling in patients with suspected meningitis.

Approximately 50% of the dry weight of the brain is attributed to its lipid content, which plays a crucial role in various biological processes due to its structural diversity. Neurolipids are vital elements of the structural membrane, essential for maintaining cellular and physiological functions in the brain (17–19). Identifying lipid biomarkers in neuroinfectious diseases could help to improve the diagnosis and deepen the understanding of the pathogenesis of the disease. In this review, we explore altered CSF metabolites in bacterial and viral meningitis, with a particular focus on those associated with disruptions in neurolipid metabolism. Thereafter, we discuss their importance in understanding their role in host, pathogen, or interaction.

## 2 Search parameters

A general online literature search was conducted for CSF metabolomics studies that used liquid chromatography-mass spectrometry (LC–MS), particularly on bacterial and viral infections of the CNS. The data bases used were Google Scholar, PubMed, and Web of Science. Online search criteria included ‘neuroinfectious diseases and LC–MS metabolomics’, ‘neuroinfectious diseases and CSF metabolomics’, ‘metabolomics and viral encephalitis/bacterial meningitis’.

## 3 Cerebrospinal fluid metabolomics of viral infections of the CNS

### 3.1 Enterovirus meningitis

Enteroviruses are a group of RNA viruses that can infect the CNS and induce acute inflammation of the brain (encephalitis) or membranes surrounding the brain (meningitis), especially in children and immunocompromised individuals (20). Enteroviral infections pose diagnostic challenges because classic indicators of viral meningitis in CSF, especially pleocytosis, which is indicative of inflammation, can appear normal in up to 15% of patients with enteroviral meningitis (21), suggesting that normal CSF findings do not exclude enteroviral meningitis. Although enteroviral RNA can be detected by PCR, there are currently no specific biomarkers for enteroviral meningitis. The identification of biomarkers could potentially lead to a more targeted and personalized approach to treatment. Currently, researchers are actively exploring the field of metabolomics to identify metabolites from the CSF that could serve as diagnostic tools for enteroviral meningitis and other viral infections of the CNS.

The metabolomics study by Ratuszny et al. (22) used a targeted LC–MS approach to identify potential CSF biomarkers for enteroviral meningitis, particularly in cases with normal CSF cell counts and compared with cases of enteroviral meningitis cases presenting with pleocytosis. The analysis revealed important metabolites that could serve as potential biomarkers for enteroviral meningitis (Table 1). Among the metabolites, kynurenine was identified as a specific biomarker for enteroviral meningitis with pleocytosis. Kynurenine is a metabolite of an amino acid tryptophan that plays a crucial role in immune regulation and neurotransmission (26). Its elevation in patients with pleocytosis suggests an increased immune inflammatory response to viral infection and warrants further investigation. Previous studies have demonstrated a close association between CSF kynurenines and inflammations, suggesting that they could serve as valuable indicators for monitoring neuroinflammation (27, 28).

**Table 1:** Altered CNS metabolites associated with CNS lipid metabolism in viral CNS infections.

CNS viral infection	Metabolite class	Metabolite	Fold change	References
Varicella zoster virus (VZV) meningitis/encephalitis	Lipids	SM (C16:1)	↑	(23)
		PC (C34:0)		
		LysoPC (C26:1)		
	Amino acid	Glycine		
Enterovirus meningitis	Lipids	PC (36:2)	↑	(22)
		PC (36:3)		
	Organic acid	Kynurenine		
Tick-borne encephalitis	Lipid peroxidation products	Malondialdehyde	↑	(24)
		4-Hydroxynonenal		
		8-Isoprostanes		
		Neuroprostanes		
	Endocannabinoids	Anandeamide		
		2-Arachidonylglycerol		
	Eicosanoids (Pro-inflammatory)	Prostaglandin E2		
		Leukotriene D4		
Herpes simplex virus	Lipid	PC.aa.C30.0	↑	(25)
		PC.ae.32.2		
		SM.C16.0		
		PC.aa.C38.0		
	Acylcarnitines	Butyrlcarnitine		
		Isovalerylcarnitine		

SM, Sphingomyelin; PC, Phosphatidylcholines; LysoPC, Lysophosphatidylcholines.

Phosphatidylcholines (PC), particularly PC 36:3, emerged as a single accurate lipid biomarker for enteroviral meningitis without pleocytosis, while PC 36:2 was associated with enterovirus meningitis, independent of pleocytosis (22). PCs constitute the majority of phospholipids in the brain (29), they are used primarily as membrane lipids, protecting cells from diverse environmental challenges and facilitating various cellular compartments (30). Alterations in PCs may be indicative of membrane damage and changes in cellular metabolism associated with viral infection. Furthermore, changes in phospholipids independent of pleocytosis in enteroviral meningitis suggest that PC alteration is not solely influenced by the inflammatory immune response; instead, pathological processes within the meninges can also play a role, and targeting these metabolites will not only aid in early detection, but also provide information on the pathogenesis of enteroviral meningitis. Changes in phospholipids have also been reported in other neurological disorders such as Alzheimer (31).

Although the findings of this study offer insights into important altered metabolic pathways in enteroviral meningitis, it is important to note that the study included a limited number of enteroviral meningitis patients (*n* = 10). Comparison was made between patients with normal cell counts and those with pleocytosis, without comparing them with other viral meningitis patients or cases of viral neuroinflammation. This makes it difficult to discern whether the findings are unique to enteroviral meningitis or common to other types of viral meningitis and neuroinflammation. Therefore, more investigation and validation studies are needed to confirm the diagnostic potential of the identified metabolites.

### 3.2 Varicella zoster virus meningitis/encephalitis

Varicella zoster virus (VZV) is an α-herpesvirus known to cause chickenpox in children, which then enters a latent state in ganglionic neurons after infection or immunization with attenuated VZV (32). Upon reactivation of the virus, triggered by various stimuli, the virus can reach the skin through anterograde spread and induce herpes zoster (shingles), characterized by a painful skin rash or blisters in most cases (33). The virus can also gain access to the CNS through retrograde spread and induce a wide range of neurological disorders. The frequently observed clinical symptoms of VZV induced encephalitis include meningoencephalitis, cerebellitus, stroke, myelopathy, altered metal status, and focal neurological (34, 35).

Cerebrospinal fluid PCR is considered the gold standard for the diagnosis of VZV-induced encephalitis with a sensitivity and specificity of 98 and 94%, respectively. However, a combination of clinical criteria and biological results is still required to confirm diagnosis, mainly because a positive CSF PCR can still yield positive results in cases of zoster without encephalitis, meaning that a positive CSF PCR alone cannot confirm the diagnosis of VZV encephalitis (36, 37). Additionally, the initial symptoms of VZV encephalitis, such as headache, fever, and altered mental status, are nonspecific and can be attributed to various other conditions, making it difficult to differentiate them from other cases of encephalitis. Current studies on VZV encephalitis are based on biological samples obtained at autopsy, but their use is also limited by the unpredictable nature of postmortem changes and the absence of small animal models to study pathogenesis at the organismal level (23). CSF metabolites are currently being explored to identify specific biomarkers for VZV encephalitis and to provide insight into the molecular mechanisms of the disease.

Kuhn et al. (23) performed a CSF-targeted metabolic screening in three manifestations of VZV reactivation, segmental zoster (*n* = 14), facial nerve zoster (*n* = 16) and zoster meningitis and/or encephalitis (*n* = 15). Zoster meningoencephalitis showed a significant association with four elevated metabolites (Table 1), which were not associated with CNS leukocyte count. Among the identified metabolites, sphingomyelin, specifically SM C16:1, exhibited the strongest association with zoster meningoencephalitis. The study also suggested that these metabolites could be specific for zoster meningoencephalitis by comparing them with the enteroviral meningitis control group. This comparison revealed a significant difference in LysoPC C26:1 between zoster meningoencephalitis and enteroviral meningitis, making this metabolite a distinguishing factor between the two groups.

### 3.3 Tick-borne encephalitis

Tick-borne encephalitis (TBE) is a viral infection of the CNS caused by the TBE virus (TBEV). It is characterized by inflammation of the brain. Although TBE is preventable by vaccination, its incidence has increased, posing a growing health concern (38). The mechanism by which TBEV breaches the BBB remains unclear. Four potential mechanisms have been proposed, including entry through peripheral nerves, olfactory neurons, transcytosis across vascular endothelial cells of the brain capillaries, and diffusion between capillary endothelial cells (39). Understanding the molecular mechanism of TBE could shed light on the pathogenesis. Tick-borne diseases have been shown to induce modifications in host lipid metabolism (24).

A recent study by Groth et al. (24) compared the antioxidant status and level of lipid mediators in the CSF of patients with TBE (*n* = 15) and patients with TBE and bacterial co-infections (*n* = 6) upon admission and after treatment. The study revealed that both TBE and TBE co-infections showed a decrease in total antioxidant status and increased levels of lipid peroxidation products, endocannabinoids, and eicosanoids before treatment (Table 1). Although co-infection with TBE showed the most significant reduction in total antioxidant status compared to only TBE, total antioxidant status did not improve after treatment in both conditions. Groth et al. suggest that a decrease in antioxidant status in the CSF may have an effect on oxidative modification of lipids. In cases of TBE co-infection, the level of malondialdehyde did not decrease after treatment. A similar trend was also observed with prostaglandin E2, which also did not decrease despite treatment.

The results of Groth et al. indicated that both TBE, a virus-induced condition, and TBE with bacterial co-infection showed similar alterations in antioxidative status and lipid mediators. However, the changes were more pronounced in patients with co-infections. The alteration of the antioxidative status is believed to contribute to disruptions in phospholipid metabolism in the CNS, as reflected in the levels of lipid peroxidation products and lipid mediators in this study. In particular, in patients with co-infections, there was a significant lack of changes in lipid mediators despite treatment. However, similar studies are needed to confirm the diagnostic potential of these metabolites and to distinguish between TBE and co-infections. These studies should also include patients with only bacterial infections to account for possible bacterial variations.

### 3.4 Herpes simplex encephalitis

Herpes simplex encephalitis (HSE) is a rare but fatal neurological disorder resulting from the infection of the CNS with the herpes simplex virus type 1 (HSV-1) or type 2 (HSV-2) virus, with HSV-1 being more prevalent than HSV-2. Like other forms of encephalitis caused by different viral infections, patients with HSE commonly experience fever, disorientation, abnormal behavior, severe headache, altered consciousness, seizures, focal neurological disabilities, and coma. However, symptoms specific to HSE often involve neurological abnormalities related to dysfunction of the fronto-temporal lobes. HSE can progress rapidly, resulting in severe symptoms and complications if not treated promptly. Therefore, it is crucial to initiate antiviral therapy (Acyclovir) immediately after suspicion of encephalitis (40, 41).

The lack of a distinct clinical presentation, such as fewer prodromal symptoms or neurological deficits in patients with HSE, makes the diagnosis of HSE difficult. Magnetic resonance imaging (MRI) scan is often performed in patients suspected of having encephalitis to better visualize the temporal lobe, which in cases of encephalitis, the MRI scan is usually abnormal. However, a normal MRI scan does not rule out HSE and the diagnosis is confirmed by the HSV PCR test (42). However, in some cases, the HSV PCR test can be misleading, as it can produce negative results in patients with encephalitis. For example, there was a case involving an elderly man who was suspected of HSE based on electrographic and imaging findings. He was promptly treated with acyclovir, but the PCR test was negative on two occasions. Consequently, antiviral treatment was discontinued, and steroids were initiated due to suspicion of autoimmune encephalitis. Unfortunately, the patient died after a few days. Subsequent autopsy confirmed encephalitis and HSV positive by immunohistochemistry (43). There have been other reports of HSE patients with negative CSF PCR (44–47). Collectively, these cases highlight the limitations of relying solely on the HSV PCR test for diagnosis and underscore the need for more effective and more specific diagnostic approaches. Researchers have now begun exploring the metabolomics field to identify diagnostic biomarkers specific to HSE.

A recent comprehensive study by Al-Mekhlafi et al. (25) used LC–MS to conduct a targeted metabolomics/lipidomics analysis of CSF samples from patients with viral CNS infections in response to HSE (*n* = 9), enterovirus (*n* = 10) and VZV (*n* = 15) and compared with autoimmune neuroinflammation and non-inflamed controls. The scree plot analysis revealed that the principal component 1 accounted for the most variance among the three groups and was mainly influenced by changes in phospholipid concentrations. Principal component 2 was influenced by differences in amino acids and, in cases of viral CNS infections versus autoimmune neuroinflammation or controls, short-chain acylcarnitines also contributed to the overall variance. High levels of acylcarnitines (C4 and C5) were observed in HSE patients than VZV and enterovirus patients. Similarly, changes in phospholipid metabolites were most significant in HSE compared to VZV and Enterovirus patients. The results of this study align with the results reported by (22, 23).

The authors suggest that changes in acylcarnitine concentration indicate possible mitochondrial dysfunction. Similarly, although all CNS viral infections showed significant alterations in phospholipid levels, indicating potential membrane disruptions during viral infection, HSE exhibited notably higher phospholipid concentrations followed by VZV while enterovirus meningitis exhibited lower concentrations of the six biomarkers. The findings of this study clearly demonstrate that HSV CNS infection induces a different host metabolic disruption, reflecting its unique pathogenic mechanisms compared to other CNS viruses. However, taken together, these results indicate membrane damage and disruption of cellular metabolism in viral CNS infections.

Herpes simplex encephalitis was also featured in a study that used a tandem mass spectrometer to measure PC.ae.C44.6 concentrations in patients with bacterial and viral infections, as well as autoimmune neuroinflammation (48). Although PC.ae.C44.6 was detected above LOD in acute bacterial meningitis and neurobollesis compared to all other groups, the study reported that the detection of PC.ae.C44.6 in HSE was significantly above LOD compared to other groups. These results support previous findings that, although encephalitis is a possible outcome of CNS viral infections in the CNS, HSV-induced encephalitis induces more pronounced metabolic changes in the host. Additionally, consistent alterations in phospholipid metabolites identified in patients with HSE warrant thorough lipid analysis as it reflects changes in lipid metabolism disruptions, this may provide insight into the pathogenesis of HSE in encephalitis and potential aid in the identification of biomarkers.

A previous study conducted by Atlas et al. (49) used HPLC-targeted analysis on patients with HSE to investigate kynurenic acid in CSF of HSE patients. The study demonstrated a significant elevation of CSF kynurenic acid in patients with HSE, particularly during the acute phase of the disease. The study also revealed a negative correlation between initial CSF kynurenine acid and the severity of long-term sequelae, suggesting a possible neuroprotective role of brain kynurenic acid, while also causing long-lasting cognitive function loss associated with the disease. The study suggests that the kynurenine pathway plays a crucial role in the regulation and neuroprotection in viral infections. The results also shed light on the complex interplay between immune response, neuroprotection, and cognitive impairment in the context of viral encephalitis.

In a separate study, researchers investigated the regulation of kynurenine and tryptophan concentrations in CSF samples from various CNS infections, including HSE (50). The findings for HSE showed that kynurenine concentrations were significantly elevated compared to noninflamed samples, with markedly higher mean values compared to enterovirus and bacterial meningitis. Furthermore, the study found that kynurenine concentrations were most positively with CSF leukocyte count, indicating a strong association between kynurenine and CNS inflammation in HSE. This is consistent with the findings of Atlas et al. (49), as both studies demonstrated a significant association of the tryptophan-kynurenine pathway in HSE. Taken together, studies on HSE highlight the affected pathways during CNS infection which include tryptophan-kynurenine, fatty acid metabolism, and lipid metabolism and a more comprehensive studies on these pathways could provide essential information on the immune pathogenesis of HSV.

### 3.5 Summary of CSF metabolomics of viral infections of the CNS

This section focused on metabolic studies, specifically LC–MS based, of viral infections of the CNS. Taken together, the findings of these studies, particularly the changes in phospholipids in different viral etiologies, underscore the significant impact on cellular lipid metabolism within the CNS. Although the specific lipid species affected differ between these infections, both highlight the interplay between viral pathogens and host lipid pathways. The elevation of phosphatidylcholine suggests a potential host response involving membrane remodeling or turnover, possibly in response to the inflammatory cascade triggered by viral infection. Elevated sphingomyelins and phospholipids in VZV encephalitis and HSE suggest a distinct alteration in sphingolipid metabolism, which may reflect a different aspect of viral pathogenesis or host immune response. Furthermore, the identification of lipid mediators in patients with TBE, particularly the presence of increased lipid peroxidation products, further underscores the disruptions in CNS lipid metabolism associated with viral CNS infections. These findings suggest that viral infections have a broader effect on neurolipid metabolism, indicating a common theme in different viral etiologies. Understanding the alterations of these pathways in viral infections could provide novel insights into the pathophysiology of viral meningitis and may reveal new therapeutic targets for the management of these infections.

## 4 Cerebrospinal fluid metabolomics of bacterial infections

### 4.1 Neurosyphilis

Neurosyphilis is a complication of untreated syphilis resulting from invasion of the CNS by the bacterium *Treponema pallidum* (51). The bacteria are transmitted through direct contact with infected lesions (such as during sexual contact) or, rarely, through blood transfusions. Following transmission, bacteria enter the body through intact or compromised mucous membranes. Once inside, it can spread through the lymphatic and bloodstream, reaching various organs, including the brain and spinal cord. In the early stages, neurosyphilis can present as asymptomatic or as meningeal neurosyphilis, where patients do not exhibit neurological symptoms but may have inflamed meninges. If left untreated, late-symptomatic neurosyphilis ensues that involves inflammation of both the meninges and the brain parenchyma (52).

Asymptomatic neurosyphilis presents with serological and CSF abnormalities presenting with pleocytosis (up to100 cells/μL) or elevated protein concentration (up to100 mg/dL) (53, 54). CSF-venereal disease research laboratory (VDRL) and CSF-rapid plasma regain (RPR) tests are considered the gold standard for the diagnosis of neurosyphilis; however, their low sensitivity of approximately 30% limits their application (55, 56) and a negative CSF-VDRL test cannot exclude the possibility of neurosyphilis (57). Currently, no single laboratory test or reliable biomarkers can rule out neurosyphilis as a diagnosis, and many asymptomatic neurosyphilis are not diagnosed (51, 56).

*Treponema pallidum* has previously been shown to persist in the body without inducing a strong immune response, making diagnosis and treatment difficult (58). The exact mechanism behind this ability is not fully understood. The slow replication cycle, which takes approximately 33 to 44 h to double, is believed to lead to a weekend immune response and milder symptoms during infection (59–61). Additionally, *Treponema pallidum*’s limited energy metabolic capacity, reflected in its smaller genome and the lack of key pathways such as those related to the tricarboxylic acid (TCA) cycle and oxidative phosphorylation, forces the bacterium to heavily depend on the host environment for essential biosynthetic functions (62). These factors contribute to the ability to persist within mammalian cells without eliciting an immune response. Understanding metabolic changes could significantly improve diagnosis and treatment.

In 2019, one of the first untargeted CSF metabolomics profiling studies compared neurosyphilis, syphilis/non-neurosyphilis and syphilis-free patients, and revealed various differential metabolites between neurosyphilis and syphilis/non-neurosyphilis or syphilis-free patients (Table 2) (63). Although there were significant differences in the identified differential metabolites between the three groups, these metabolites, particularly D-mannose, L-gulono-gamma-lactone and N-acetyl-L-tyrosine, were present in comparisons of the three groups. The alterations in these metabolites were not only specific for neurosyphilis, but are common in all groups. A separate profiling study would be necessary that includes other neurological disorders and patients would be necessary to determine the specificity of these metabolites.

**Table 2:** CSF altered metabolites associated with lipid metabolism in CNS bacterial infections.

CNS bacterial infection	Metabolite class	Metabolites	Fold change	Reference
Neurosyphilis	Purine	Hypoxanthine	↓	(63)
	Carbohydrates	L-Gulono-gamma-lactone		
		D-Mannose		
	Amino acid	N-Acetyl-L-tyrosine	↑	
	Lipid	Prostaglandin E2	↓	(56)
		Alpha-kamlolenic acid		
	Acylcarnitine	Butyryl-L-carnitine	↑	
		Palmitoyl-L-carnitine		
	Amino acid	L-Histidine	↑	
	Dicarboxylic acid	Bilirubin	↑	
Tuberculous meningitis	Lipids	Lathosterol	↑	(64)
		Phosphatidic acid		
		Phosphatidylcholine		
		Phosphatidylglycerol		
	Fatty acids	Linolenic acid	↑	
		Palmitic acid		
		16-Hydroxypalmitic acid		
		Stearic acid		
	Dicarboxylic acid	Bilirubin	↑	
	Amino acid	Tryptophan	↓	(65)
	Organic acids	Kynurenine	↑	(27)
		Kynurenic acid		
		Quinolinic acid		
	Organic acid	Kynurenine	↑	(66)
	Amino acid	Tryptophan	↓	
	Acylcarnitine	Carnitine	↑	

However, hypoxanthine was notable for its significant decrease in neurosyphilis patients compared to syphilis/non-neurosyphilis patients. Hypoxanthine is a naturally occurring purine derivative involved in the purine salvage pathway and has been associated with brain edema in patients with large ischemic stroke (67). In another study, hypoxanthine was shown to interfere with lipid metabolism by inducing cholesterol accumulation through the induction of reactive oxidation species (ROS) in hepatic cells (68). Its decrease in neurosyphilis patients is unknown and warrants further investigation.

Following the untargeted metabolic profile, a targeted metabolic analysis of CSF was performed on neurosyphilis (*n* = 15) and non-neurological (*n* = 14) samples using LCMS. Various metabolites were found to differentiate the two groups (Table 2) (56). Alpha-kamlolenic acid stands out as a unique hydroxylated polyunsaturated fatty acid of C18 that presents itself as a triacylglycerol estolide (TAG-estolide) (69). Although there is relatively limited information on alpha-kamlolenic acid, polyunsaturated fatty acids are known to modify the gene expressions of lipid-lipoprotein metabolism. Deficiency in polyunsaturated fatty acids has been associated with significantly reduced concentrations of very low density lipoprotein (VLDL) triacylglycerol and synthesis of apolipoprotein B (70, 71). Qi et al. (56) linked reduced levels of alpha-kamlolenic acid in neurosyphilis to an increased susceptibility to hyperlipidemia.

Prostaglandins are lipid mediators involved in inflammatory responses, particularly pathogenic neuroinflammatory diseases (19). Reduced prostaglandin levels in neurosyphilis are suggested to be influenced by their transport across the blood-CSF barrier, which is a cerebral clearance system for prostaglandin E2 produced in the brain (56). Prostaglandin overproduction plays a role in the progression of bacterial infection-induced neuroinflammation and can contribute to brain tissue damage (72). Therefore, cerebral prostaglandin clearance is a regulatory mechanism that contributes to reducing inflammation and limiting further damage to tissue damage in the brain.

Elevated levels of acylcarnitines, especially significantly increased palmitoylcarnitine in neurosyphilis, suggest potential disruptions in energy metabolism and mitochondrial dysfunction. Palmitoylcarnitine is a long-chain acylcarnitine that facilitates the transport of long-chain fatty acids into mitochondria for beta-oxidation. Although short-chain fatty acids can enter mitochondria directly, long-chain fatty acids require active transport involving complex enzymatic reactions and regulatory processes (73). The elevation of extracellular long-chain acylcarnitines may result from the body utilizing short-chain fatty acids, which are readily available, to meet increased energy demands possibly caused by bacterial infection in the central nervous system. Qi et al. suggest that the presence of acylcarnitines in neurosyphilis may be associated with the risk of cardiovascular diseases (56).

### 4.2 Tuberculous meningitis

Tuberculous meningitis (TBM) is the most severe manifestation of extrapulmonary tuberculosis. Globally, tuberculosis ranks as the second leading cause of mortality attributed to a single infectious agent worldwide, after COVID-19 (74–76). According to World Health Organization assessments, approximately 10.6 million people developed pulmonary tuberculosis (TB) in 2021, an increase of 4.5% from 10.1 million in 2020 (World Health Organization, 2022). Individuals with TB can develop a severe complication known as tuberculous meningitis (TBM), which accounts for approximately 1% of all active cases of TB (77, 78). Young children (<5 years old), adults with HIV coinfection and immunocompromised individuals represent a significant potent risk factor for TBM (79). Despite ongoing research and improvements in diagnostic and therapeutic approaches, TBM remains a major global health problem.

Primary infection by *Mycobacterium tuberculosis* usually leads to pulmonary TB upon inhalation of the bacteria. From there, the bacteria can disseminate to the CNS through the lymphatic system or the bloodstream (80). The development of TBM is believed to occur when *Mycobacterium tuberculosis* spreads from the lungs to the brain parenchyma, forming granulomas that later rupture into the leptomeninges, resulting in TBM (81). Recent studies have shown that chronic granulomatous inflammation is initiated almost exclusively in the leptomeninges and not in the brain parenchyma (82, 83). The pathogenesis of TBM continues to be a topic of ongoing debate.

TBM presents challenges in diagnosis due to the nonspecific nature of its symptoms, the absence of rapid and sensitive diagnostic tests, and challenges in detecting bacteria in CSF (77). Universally accepted diagnostic standards for all types of meningitis involve the analysis of CSF obtained by lumbar puncture. Traditional CSF markers for TBM include strongly elevated protein levels, reduced glucose levels, and increased WBC, predominantly lymphocytes (9). CSF culture is necessary for a definitive diagnosis, but it may not always detect *Mycobacterium tuberculosis* in small volumes (84, 85). Brain magnetic resonance imaging (MRI) is widely considered the most sensitive and reliable method of diagnosing CNS disorders; however, its availability is limited in low-income or resource-poor countries (84). Despite significant progress in diagnostic tools for TBM, there is still a need for rapid, precise, and cost-effective diagnostic tests.

Researchers have ventured into the realm of metabolomics to look for distinctive biomarkers for TBM, given the indication of metabolic changes and dysregulation of the immune response in TBM. In 2017, an untargeted metabolomics profiling study was conducted on CSF samples from TBM (*n* = 50), viral meningitis (*n* = 17), bacterial meningitis (*n* = 17) and cryptococcal meningitis (*n* = 16) patients (64). The study reported six major altered metabolic pathways that distinguish TBM from other types of meningitis, including fatty acids and lipid metabolites (Table 2). Most altered metabolites are associated with energy metabolism, reflecting the increased energy demand in TBM. The study also noted an increase in the synthesis and degradation of ketone bodies, which may be linked to changes in lipid metabolites. These alterations appear to be a response to the elevated energy requirements in the TBM. The body resorts to the use of ketone bodies as an alternative energy source during excess energy demand. The limitation of the study was the absence of control groups.

In another study, CSF metabolites were measured in adult with TBM (*n* = 33), including those who died in the hospital, as well as in control subjects (*n* = 22) (65). Tryptophan was particularly notable for its significant alteration, showing markedly lower concentrations in patients with TBM who survived compared to patients who died (9 times less) and the control group (31 times less). These findings indicate that CNS infection with *Mycobacterium tuberculosis* has a profound impact on tryptophan metabolism (65). About 95% of the amino acid tryptophan is metabolized to kynurenine, where it generates several neuroactive metabolites, including kynurenic acid, 3-hydoxykynurenine and quinolinic acid (86, 87). These metabolites are known to possess anti-inflammatory properties and may exhibit immune regulatory properties that could alleviate the response to inflammation (26). The study by van Laarhoven et al. (65) is one of the pioneering works that demonstrated the significance of tryptophan in TBM. It laid the foundation for further research in this area, highlighting the potential role of tryptophan metabolism in the pathogenesis of TBM.

In 2023, a targeted metabolomics analysis was performed in adults with TBM to understand how tryptophan metabolism is altered in patients with TBM (27). The study investigated the association between CSF tryptophan levels and mortality in patients with TBM and the findings confirmed that increased CSF tryptophan is indeed associated with the mortality of patients with TBM. The study also explored the relationship between CSF tryptophan levels and CSF mycobacterial load and discovered that tryptophan was higher in CSF cultures negative and lower in CSF cultures positive, suggesting that CSF tryptophan levels may be influenced by the presence or absence of mycobacteria in TBM. Low levels of tryptophan in CSF culture positive and high levels in CSF culture negative could be attributed to the utilization of host tryptophan for its metabolic needs needed for its survival and growth within the host environment.

The study by Ardiansyah et al. also revealed an increase in the downstream metabolites of CSF tryptophan (Table 2), these metabolites are usually increased during an inflammatory immune response and are known to exhibit antibacterial properties and may influence the immune response (88, 89). The increase in kynurenine metabolites seems to be due to neuroinflammation rather than increased conversion of tryptophan to kynurenine. This is also supported by the study by Sühs et al. (50), who reported significantly lower concentrations of kynurenine in non-inflamed or mildly inflamed CSF samples compared to inflamed samples, particularly in cases of bacterial meningitis, HSE and neuroborrelosis.

Overproduction of downstream tryptophan-kynurenine metabolites could also play a role in the pathogenesis of TBM, as quinolinic acid has been shown to induce lipid peroxidation in rat brain homogenates and rat corpus striatum (90–92), indicating a potential contribution of this metabolite to energy disruption in TBM. The relationship between CSF tryptophan, neuroinflammation, kynurenine metabolites, and the regulation of indoleamine 2,3-dioxygenase (IDO) expression requires further investigations to provide novel insights into the pathogenesis of TBM (93).

A recent study used an integrated approach (metabolomics and cytokine profiling) to investigate the relationship between altered metabolites and cytokines in TBM, providing valuable information on the association between metabolic disturbances and inflammation. The study revealed severe disruptions in energy metabolism and alteration of multiple metabolites in TBM, including carnitines and tryptophan (Table 2). Ketone bodies arise from mitochondrial beta oxidation, a process contingent on the availability of carnitines (94, 95) and are used as an alternative energy source during starvation or increased energy demand in the brain. Changes in energy metabolism and the observed increase in carnitine levels in this study may signify increased energy requirements for the brain. Carnitines are actively involved in the regulation of metabolism, suggesting that their levels are influenced by metabolic processes, and dysregulation can affect overall energy metabolism.

The elevation of CSF kynurenines and reduced CSF tryptophan confirms the finding of Ardiansyah et al. (27) that demonstrated an inverse correlation between CSF tryptophan and downstream tryptophan-kynurenine metabolites. In the same study, numerous elevated pro-inflammatory cytokines (interferon (IFN)-γ, interleukine (IL) -15, and IL-2) were associated with confirmed TBM, suggesting that these mediators are active in cases of TBM with higher bacterial burden. Based on these findings and those reported by Ardiansyah et al. (27), it can infer that elevations of CSF kynurenines are independent of the status of CSF tryptophan in TBM and that CSF tryptophan appears to be more related to the pathogenesis of TBM itself than being a consequence of the immune response.

The studies discussed in these reviews advocate for a deeper exploration of tryptophan-kynurenine metabolism, fatty acid metabolism and lipid metabolism in both viral and bacterial infection. Tryptophan metabolism and its kynurenine metabolites appear to be significantly altered in bacterial infection compared to viral infections, but the underlying mechanism of how tryptophan metabolism is altered in TBM and other bacterial infections remains unknown (96). A deeper investigation of tryptophan metabolism could shed light on how tryptophan metabolism is altered in TBM and its association with neuroinflammation, providing a comprehensive understanding of the pathogenesis of TBM. Similarly, carnitine alterations in bacterial infections suggest further investigation. Targeted acylcarnitine research on TBM could provide information on how carnitines are altered and their effect on TBM. Lipid metabolites have also been identified and altered in both viral and bacterial infections, suggesting further exploration of lipid metabolism. There is a notable absence of neurolipidomics studies despite the numerous reported changes in metabolites related to lipid metabolism and the inherent lipid-rich nature of the brain. The introduction of neurolipidomics studies has great potential to improve the diagnosis of CNS diseases.

## 5 Neurolipidomics in bacterial and viral infections

Neurolipidomics is increasingly gaining significant attention in the study of CNS-related diseases. Lipidomics emerges as a promising and highly relevant field for investigating lipid biomarkers and their associated pathways in neuroinfectious diseases. This interest is fueled by compelling evidence from metabolomics studies that reveal disruption of neurolipid metabolism during CNS infections by pathogens. Disruptions in lipid metabolism have also been reported in other metabolic disorders, aging, and neurodegenerative diseases (97). The importance of lipids in the brain is highlighted by the various biological functions of brain lipids that include membrane formation, cell signaling, energy storage, neurotransmitter release and, crucially, maintaining homeostasis (98). Alterations in these lipids have detrimental consequences for patients. Therefore, the study of brain lipids holds promise to advance the diagnosis and prognosis of neurological diseases. Currently, there are limited CSF lipidomics studies in neuroinfectious diseases.

The most related study used LC–MS-targeted lipid mediator profiling to investigate mechanisms driving the inflammatory response in TBM (99). The study used a lipid mediator profiling approach to investigate the regulation of a novel group of host protective mediators (specialized pro-resolution mediators) in the CSF of adults with TBM before and during treatment. They reported that before treatment, the severity of the disease was associated with concentrations of mediators of inflammatory and pro-resolution, with more severe disease linked to lower concentrations of SPM and higher concentrations of immunosuppressive, vasoconstrictive and nociceptive eicosanoids. Researchers further grouped CSF lipid mediators by function to study their relationship with TBM severity and found that pro-resolving concentrations decreased with increasing disease severity. A previous study by Tobin et al. (100) on zebrafish and humans demonstrated an interplay between eicosanoids, tumor necrosis factor (TNF)-α and leukotriene A4 hydrolase (LTA4H) in regulating the inflammatory response to mycobacterial infection, suggesting further research to understand how these components interact and influence the immune response in diseases.

Lipidomics studies in tuberculosis have shown that infection with *M. tuberculosis* alters host lipid metabolism by significantly lowering levels of total cholesterol, triglycerides, high-density lipoprotein, and low-density lipoprotein in TB patients compared to control groups. The study suggests that low lipid levels may be associated with the severity of inflammation in patients with TB (101). Although it is known that TB can worsen malnutrition, leading to weakened immunity and potentially contributing to the progression of latent TB to active TB, it remains unclear whether low lipid profiles precede the progression of the disease or if active TB itself causes low lipid profiles (102, 103). A similar study profiled the lipidome of TB patients and showed that after treatment, cholesterol ester, monoacylgycerols and phosphatidylcholine were up-regulated, while triglycerols, sphingomyelin, and ether-linked phosphatidylethanolamine were down-regulated (104). Although these studies were conducted on TB patients and not TBM or other neuroinfectious diseases. Both revealed the alteration of host lipid metabolism after infection with *M. tuberculosis*, the same bacteria that causes TBM.

In another study comparing CSF metabolite concentrations between patients with bacterial meningitis/encephalitis and noninflamed controls, phosphatidylcholines were identified as sensitive biomarkers for bacterial meningitis due to their significant elevation compared to both the control and viral meningitis groups (16). Based on these findings and those of the studies on viral and bacterial CNS infections discussed in this review, future targeted metabolic exploration of fatty acid metabolism, tryptophan metabolism, lipidomics, and related pathways in both types of infections is warranted to explore their potential as diagnostic or therapeutic targets.

## 6 Summary and future prospects

Metabolic profiling of neuroinfectious diseases has gained significant attention over the years to understand their pathogenesis and discover potential biomarkers that could facilitate the early diagnosis of these diseases. However, despite persistent efforts and various metabolic profiling studies using different analytical techniques, there remains a lack of specific biomarkers capable of distinguishing different types of meningitis/encephalitis. There is also a notable lack of metabolic and lipidomic studies in CSF from children with TBM. However, metabolomics has provided information on significant alterations in metabolic pathways, which offers the potential to identify specific biomarkers.

The evidence presented in this review suggests a possible disruption of fatty acid metabolism, tryptophan metabolism and lipid metabolism, mainly through alterations in acylcarnitines, kynurenines, and phospholipids in both in viral and bacterial infections. Further studies on these metabolites and their associated pathways could potentially aid in the identification of important biomarkers and improve diagnosis. The kynurenine pathway is of high importance in both bacterial and viral infections and deserves a more comprehensive investigation, as it includes the enzymes involved and all kynurenine products. Furthermore, exploring the lipidome of the CNS holds promise in advancing a more comprehensive understanding of CNS-related diseases. Modified metabolites (e.g., acycarnitines) play an important role in modulating lipid metabolism in the CNS, and vice versa. Based on our analysis, this review underscores the importance of using existing metabolic data and the need to initiate targeted neurolipidomics studies. Together, these data could provide a powerful means of elucidating the precise functions of pathogenesis in neuroinfectious diseases and potentially uncover specific biomarkers.

## Author contributions

OP: Writing – review & editing, Writing – original draft, Investigation, Formal analysis, Data curation. AF: Writing – review & editing, Supervision. MK: Writing – review & editing, Supervision. SM: Writing – review & editing, Supervision, Conceptualization.
